# Green synthesis and antibacterial effects of aqueous colloidal solutions of silver nanoparticles using camomile terpenoids as a combined reducing and capping agent

**DOI:** 10.1007/s00449-016-1599-4

**Published:** 2016-04-15

**Authors:** Magdalena Parlinska-Wojtan, Małgorzata Kus-Liskiewicz, Joanna Depciuch, Omowunmi Sadik

**Affiliations:** Institute of Nuclear Physics, Polish Academy of Sciences, 31342 Kraków, Poland; Biotechnology Centre for Applied and Fundamental Sciences, Department of Biotechnology, University of Rzeszow, Sokołowska Street 26, 36-100 Kolbuszowa, Poland; Department of Chemistry, State University of New York at Binghamton, Binghamton, NY 13902 USA

**Keywords:** Green synthesis, Silver nanoparticles, Camomile, Antibacterial properties

## Abstract

Green synthesis method using camomile extract was applied to synthesize silver nanoparticles to tune their antibacterial properties merging the synergistic effect of camomile and Ag. Scanning transmission electron microscopy revealed that camomile extract (CE) consisted of porous globular nanometer sized structures, which were a perfect support for Ag nanoparticles. The Ag nanoparticles synthesized with the camomile extract (AgNPs/CE) of 7 nm average sizes, were uniformly distributed on the CE support, contrary to the pure Ag nanoparticles synthesized with glucose (AgNPs/G), which were over 50 nm in diameter and strongly agglomerated. The energy dispersive X-ray spectroscopy chemical analysis showed that camomile terpenoids act as a capping and reducing agent being adsorbed on the surface of AgNPs/CE enabling their reduction from Ag^+^ and preventing them from agglomeration. Fourier transform infrared and ultraviolet–visible spectroscopy measurements confirmed these findings, as the spectra of AgNPs/CE, compared to pure CE, did not contain the 1109 cm^−1^ band, corresponding to –C–O groups of terpenoids and the peaks at 280 and 320 nm, respectively. Antibacterial tests using four bacteria strains showed that the AgNPs/CE performed five times better compared to CE AgNPs/G samples, reducing totally all the bacteria in 2 h.

## Introduction

Nanoscience allows fabricating particles with diameters between 1 and 100 nm [1], which can be used in materials science, chemistry, medicine or biology. In the two latter segments, one of the most popular nanoparticles (NPs) is silver particles [2], as they show great antibacterial and anticancer activities [3–7]. The unique properties of these nanoparticles are determined by their small size (1–100 nm), but also their shape, structure and surface functionality [8]. Various manufacturing methods of silver nanoparticles are known, but most of them are harmful for the environment, because they require the use of heavy and toxic chemicals, which are necessary in chemical and photochemical reactions and microwave assisted processes [9–11]. Green synthesis involves a set of principles to reduce or eliminate the use or generation of hazardous substances in the design, manufacture and application of silver nanoparticles [12, 13]. Thus, synthesis methods based on the use of plant extracts [13] to synthesize the silver nanoparticles from silver nitrate are of great interest.

The use of plant parts and isolated phytochemicals for the prevention and treatment of various health disorders has been in practice for many decades. For the treatments, bioactive components from medicinal plants, which are similar to chemical compounds, are used [14, 15]. Extracts of plants, used in green synthesis, include active molecules acting as reducing and capping agents such as flavonoids, tannins, amines, aldehyde/ketone groups and polyols and proteins for Ag^+^ [16–18]. Syntheses of silver nanoparticles using black pepper leaf extract [19], olive leafs [20], cinnamon barks [21], grape seed extract [22], or papaya fruit extract [23] have been reported.

Many studies have described the beneficial properties of camomile herb (*Matricaria camomilia*), which is one of the oldest and the most popular plants used in medicine [24, 25]. Camomile has been used to treat various inflammations, wounds, neuralgia, ulcers and rheumatic pains [26, 27] or as mouthwash to treat gingivae [28]. Moreover, the extract of camomile inhibits the growth of human cancer cells [29]. The therapeutic activity of camomile is due to different effective substances such as phenolics and flavonoids apigenin, quercetin, patuletin, luteolin, and their glucosides. Other compounds in camomile include: sesquiterpenes, terpenoids, flavonoids, coumarins such as herniarin and umbelliferone, phenylpropanoids such as chlorogenic acid and caffeic acid.

The aim of this study is to eliminate heavy chemicals in the design and manufacture of silver nanoparticles (AgNPs) from aqueous extracts of camomile for antimicrobial applications. In addition, by synthesizing in camomile extract it is expected to achieve a synergistic cytotoxic effect. These green-synthesized silver nanoparticles in camomile extract (AgNPs/CE) were examined by scanning transmission electron microscopy (STEM), energy dispersive X-ray analysis (EDX), Fourier transform infrared spectroscopy (FTIR) and UV–vis to determine, which compound of camomile reduces Ag^+^ ions into Ag nanoparticles. The cytotoxic effect of AgNPs/CE was analyzed by the percentage of reduction, the zone of inhibition and the minimum inhibitory concentration on four different bacteria strains, and the results were compared to conventionally synthesized Ag NPs.

## Experimental

### Synthesis of AgNPs

Silver (I) nitrate (AgNO3, ≥99.0 %) and glucose (C_6_H_12_O_6_ ≥99.5 %) were purchased from Sigma-Aldrich, Poland. The silver nanoparticles were directly synthesized in the camomile extract via a simple green synthesis procedure. The camomile extract was prepared by mixing 500 ml of deionized water and 100 g of dried camomile flowers and keeping the obtained slurry at 90 °C for 5 h without boiling. After cooling, the extract was filtered using filter paper. Next, 5 ml of the flower extract was mixed at room temperature with 500 ml of silver nitrate solution (1 mmol). The obtained mixture was kept undisturbed in a dark place. With time, the color of the solution changed into a reddish-brown, which was associated with the formation of silver nanoparticles. Finally, the NPs were collected by centrifugation. The concentration of the used camomile extract was 0.167 mg camomile extract/1 ml. As the synthesis of AgNPs/CE was successful at room temperature, further effects of camomile extract concentration or the influence of temperature on the formation of Ag nanoparticles were not performed.

The pure (without any plant extracts) Ag nanoparticles, used as a reference sample to evaluate the antibacterial properties of the camomile synthesized silver NPs, were synthesized using the following procedure. 50 ml of glucose solution (0.03 M) was mixed with 100 μl of silver nitrate (0.15 M) at room temperature. Subsequently, it was kept undisturbed in dark place for 2 h until it changed the color to gray. To simplify further sample nomenclature, the initial concentration of silver nitrate in AgNPs/CE (300 µM) was used to estimate the concentrations for minimum inhibitory concentration (MICs) evaluation.

### Structural characterization

The structure of the pure camomile extract (CE), camomile extract with silver nanoparticles (AgNPs/CE) and pure silver nanoparticles synthesized with glucose (AgNPs/G) were analyzed by transmission electron microscopy (TEM). A drop of each suspension was deposited on a TEM Cu grid coated with a carbon foil, and subsequently dried. The sample was observed in the scanning transmission electron microscopy (STEM) mode using the high angular annular dark field (HAADF) detector on a FEI Tecnai Osiris operating at 200 kV with a resolution of 0.15 nm in STEM. The FEG instrument is equipped with a unique in-column EDX system allowing for fast acquiring of EDX maps of the sample.

### Infrared spectroscopy measurements

The FTIR spectroscopy measurements were performed using the Vertex 70 (Bruker) spectrometer employing the attenuated total reflectance (ATR) technique; a diamond crystal was applied for this purpose. All the analyses were performed in the average IR range, more precisely, between 400 and 3500 cm^−1^ wavelength. 64 scans were used to obtain a spectral resolution of 4 cm^−1^.

### UV–vis

The UV–vis measurements were performed with an evolution 3000 instrument from Thermo Scientific for all three suspensions. The resolution was chosen to be 2 nm and the scan speed was 240 nm/min. The spectral range was from 250 to 900 nm.

### Particle size measurements

The particle size measurements were performed using a Zetasizer Nano ZS instrument from Malvern. Dynamic light scattering (DLS) technique is used to measure the particle size. The measurements were performed at room temperature (25 °C) using red laser (633 nm, 4 mW) and the measurement angle was set at 173°.

### Antibacterial properties

#### Bacterial strains and cultures medium

Four bacteria strains were employed to evaluate the potential of antibacterial activity of green synthesized silver nanoparticles. Gram-positive (*Staphylococcus aureus* ATCC 25923, *Bacillus subtillis* PCM 486) and gram-negative (*Pseudomonas aeruginosa* ATCC 27853, *Escherichia coli* PCM 2209) bacteria were studied. Nutrient broth (NB) or nutrient agar (NA) was used for the preparation of bacterial cultures suspension or evaluation of the colony forming unit (CFU), respectively. All media were purchased from the BTL Company (Poland). For serial dilutions phosphate buffered saline (1×PBS; 137 mM NaCl, 2.7 mM KCl, 10 mM Na_2_HPO_4_, 1.8 mM KH_2_PO_4_) was used.

#### Antibacterial properties

The camomile extract (CE), camomile extract synthesized silver nanoparticles (AgNPs/CE) and pure silver nanoparticles synthesized with glucose (AgNPs/G), were tested for their antibacterial activity. Three different experiments were conducted: (1) percentage of reduction, (2) zone of inhibition and (3) minimum inhibitory concentration (MIC) of tested strains according to the standard protocols Clinical and Laboratory Standards Institute (CSLI ), 2012. The bacteria cultures were prepared in 10 ml of NB followed by overnight shaking at 37 °C. (1) The initial inhibitory effects on the growth of microorganisms were tested against the *S. aureus* strain. After the exposure to the tested nanosuspensions (CE, AgNPs/CE and AgNPs), the bacteria colonies were counted and the CFU/ml was estimated (the flowchart on Fig. 5c). Before the experiment, the density of the overnight inoculum was estimated and diluted to absorbance of 0.1 OD_600_, which corresponds to 10^6^ cells/ml. Each of the tested microorganisms was mixed (in a ratio 1:9) with the CE, with AgNPs/CE and with AgNPs/G, and subsequently incubated at 37 °C with shaking. The sampling time for the experiment was 0, 4 and 24 h. After the exposure period, bacteria colonies were counted and CFU were calculated. In the next step, the percentage of reduction (%*R*) in the growth of bacteria was estimated, however, in a shorter time regime. Samples were plated in triplicate, and the counts on the three plates were averaged. The inactivation efficiency (%*R*) was calculated according to formula:

$$\% R = \left( {\frac{{{\text{CFU}}_{\text{control}} - {\text{CFU}}_{\text{sample}} }}{{{\text{CFU}}_{\text{control}} }}} \right) \times 100\;\% .$$

(2) The zones of inhibition were measured according to a modified standard protocol [30]. This modification consisted in punching 5-mm diameter wells in the nutrient agar instead of using a soaked disc. This modification is motivated by the difference in the diffusion rate of the substances through the agar. The latter one depends on the depth, as well as on the molecular weight of the substances. 100 µl (10^8^ CFU/ml) of bacterial suspension was spread onto the agar plates. When dried, 50 µl of AgNPs/CE or pure AgNPs/G were aseptically transferred into separate wells. The plates were incubated at 37 °C for 24 h and the average diameter of the inhibition zone surrounding the wells was measured. All tests were done in triplicate; the mean and standard deviations were estimated. (3) The broth microdilution method was used to determine the lowest concentration of the tested camomile and silver NPs containing solutions. The MIC assay was done according to the standard protocols [30] and previously cited in [31, 32]. Four tested strains were grown overnight on nutrient broth, and then were diluted to match the absorbance ~0.1 (OD_600_, TECAN spectrophotometer), which corresponds to 1.5 × 10^6^. 100 µl of cells suspensions were distributed to the 96-well microtiter plate. Two of the tested nanosuspensions, AgNPs/CE and AgNPs/G, were diluted, and 100 µl of each dilution were added to the wells containing bacterial cells. The negative (pure medium) and positive (pure medium with bacteria) controls were maintained. The microtiter plates were incubated for 24 h at 37 °C with shaking (250 rpm). The lowest concentration of the tested nanosuspensions, which was transparent, was considered as the MIC.

## Results

### Particle size distribution

**Fig. 1:**
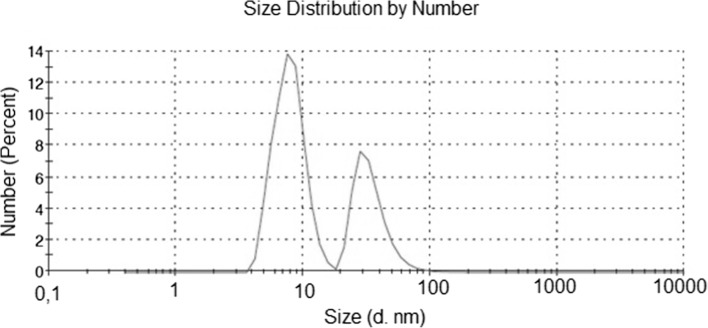
DLS spectrum of size distribution by number for Ag nanoparticles synthesized in the camomile extract

Global information about the particle size distribution of the AgNPs/CE was obtained from the DLS measurements shown in Fig. 1. Two peaks are visible, showing an average particle size of 8 and 35 nm, respectively. The peak at 8 nm is much more intense reaching 66.4 % compared to the 35 nm peak at 33.6 %. To explain the nature of the particles generating these two peaks, transmission electron microscopy was applied to have more detailed local and structural information.

### Structural characterization

**Fig. 2:**
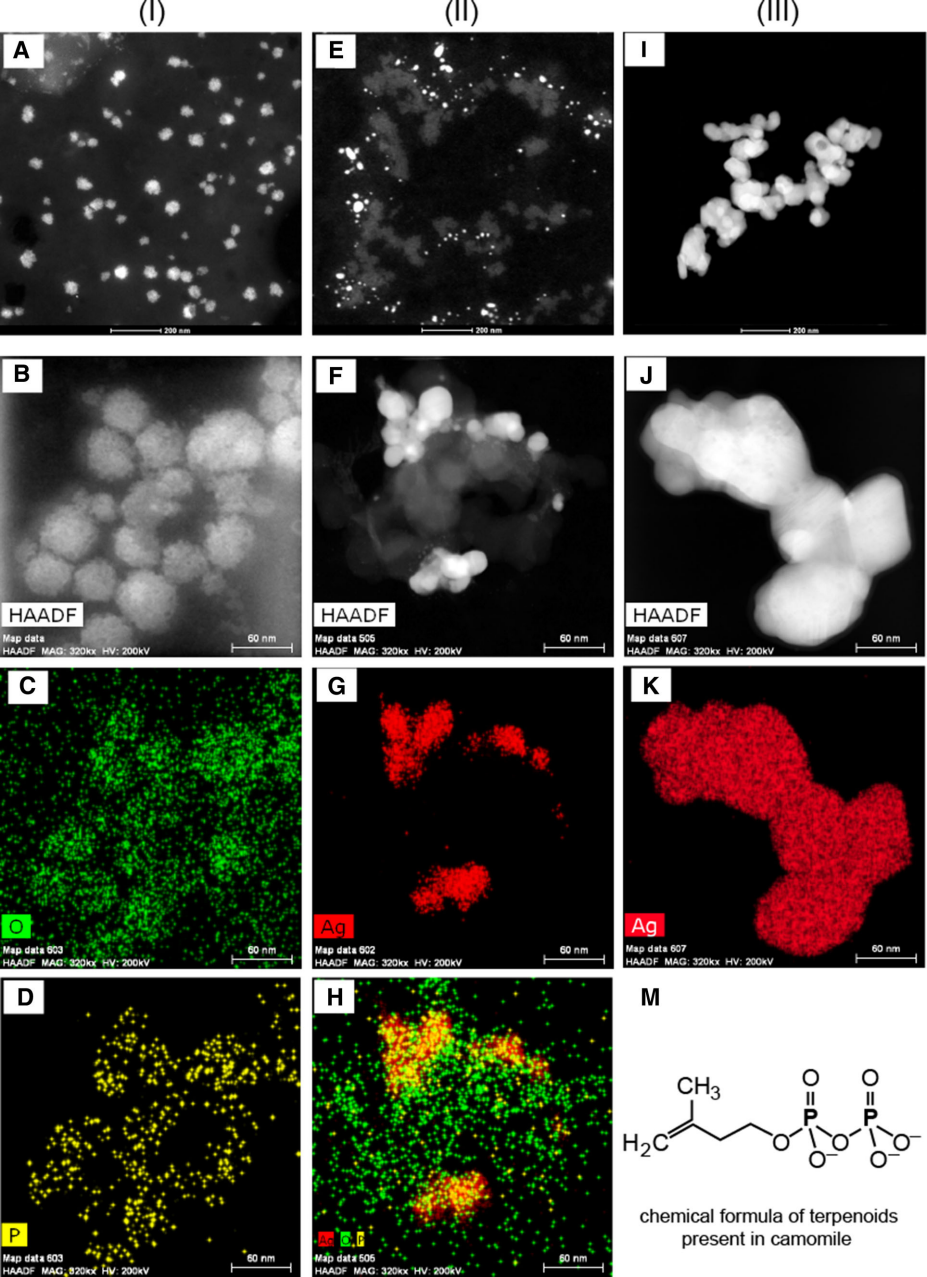
STEM HAADF image with the corresponding EDX maps of I pure camomile extract used for the synthesis; II Ag particles synthesized in the camomile extract; III Ag particles synthesized with glucose; M chemical formula of terpenoids showing the presence of phosphorus

Thus, the structures of the CE, the AgNPs/CE and AgNPs/G were imaged by electron microscopy. Figure 2 presents the combination of STEM HAADF images with the corresponding quantified EDX maps of pure camomile extract (column I), Ag particles synthesized in the camomile extract (column II) and pure Ag particles synthesized with glucose (column III). The camomile extract consists of globular structures, which are agglomerated forming porous aggregates and networks. The spherical globules have sizes varying from approximately 20 to 70 nm, Fig. 2a, b, what explains the presence of the DLS peak at 35 nm average particle size, Fig. 1. In the quantified EDX maps of the camomile extract the distribution of phosphorus and oxygen is shown, Fig. 2c, d. Phosphorus is an element present in terpenoids, Fig. 2m, a component of camomile. In the AgNPs/CE sample, column II in Fig. 2, the HAADF STEM images showed Ag particles uniformly distributed on the camomile globules. In the overview and the magnified images, Fig. 2e, f, respectively, the camomile extract is visible as a light gray contrast in between the Ag nanoparticles, however, the globules are more agglomerated then in pure camomile extract. The Ag particle size scattering is relatively large reaching from 2 to 25 nm, what corresponds to the average particle size of 8 nm measured by DLS, Fig. 1. The quantified EDX maps clearly show the presence of Ag, oxygen and phosphorus originating from the camomile extract. Interestingly, phosphorus is not anymore detected on the camomile globules, but rather on the AgNPs. The structure of Ag nanoparticles synthesized without camomile extract is presented in the overview and the larger magnification images in column III, Fig. 2i, j, respectively. Due to the same scale bar it can be clearly seen that the AgNPs/G synthesized without camomile are much more agglomerated and much larger compared to AgNPs/CE. The quantified contents in at. % of oxygen, phosphorus, carbon and silver for the CE and AgNPs/CE samples are given in Table 1.

**Table 1:** Quantified contents estimated by EDX of oxygen, phosphorus, carbon and silver for the CE and AgNPs/CE samples

	O (at%)	C (at%)	P (at%)	Ag (at%)
CE	31.5	64.7	3.9	–
AgNPs/CE	7.2	69	1.4	22.5

**Fig. 3:**
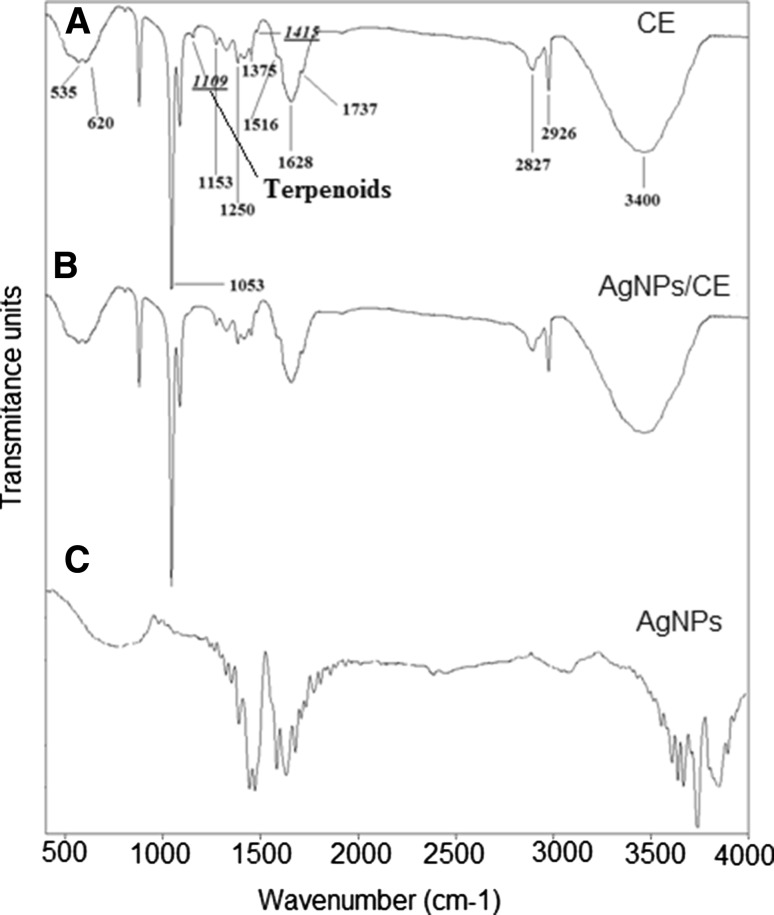
Typical FTIR absorption spectra of: *a* pure camomile extract CE and *b* camomile extract synthesized silver nanoparticles AgNPs/CE, *c* AgNPs/G synthesized by the Ag^+^ reduction with glucose

Figure 3a shows the FTIR spectrum of pure camomile extract CE. The band between 3410 and 3370 cm^−1^ matches the O–H stretching in hydroxyl groups corresponding to phenol, and the N–H stretching corresponds to amides. The peaks between 2920 and 2850 cm^−1^ are characteristic for the C–H vibration (stretching) of aliphatic groups from glucosides. The band between 1750 and 1620 cm^−1^ corresponds to the C=O vibration of bonded, conjugated esters from luteolin. The band at approximately 1640 cm^−1^ is attributed to the C=C vibration of the aromatic chain in carboxylic acids probably from patulin. The peak around 1320–1370 cm^−1^ originates from the C–N stretch vibration of the aromatic amine I and amine II. The band around 1250 cm^−1^ corresponds to C–H stretching and O–H deformation of carboxyl groups and to the N–H bond of amide II. The peak at 1109 cm^−1^ is attributed to the –C–O group of terpenoids [33]. The broad band centered around 1050 cm^−1^ is attributed to aromatic ethers and polysaccharides (C–O–C stretching). The bands at 900–600 cm^−1^ correspond to primary and secondary amines and amides [34]. The FTIR spectrum of AgNPs/CE nanoparticles, compared to pure CE, does not contain the band at 1109 cm^−1^, which corresponds to –C–O groups of polyols such as flavones, terpenoids and polysaccharides, Fig. 3b. These polyols are mainly responsible for the reduction of silver ions [35]. Figure 3c shows the FTIR spectrum of AgNPs/G synthesized by Ag^+^ reduction with glucose. The vibrational spectra can be divided into two main groups of peaks. The region from 1000 to 1700 cm^−1^ where C–O and C–C groups vibration modes are present and the carbohydrates generally show their characteristic bands. The second region contains bands from 2900 to 3450 cm^−1^ assigned to CH and OH vibrations groups [36]. All these peaks are summarized in Table 2.

**Table 2:** FTIR analysis of camomile extract and AgNPs/CE

Wavenumber (cm^−1^)	Camomile extract (CE)		AgNPs/CE	
	Bond/stretching	Functional group	Bond/stretching	Functional group
535	C–N stretch	Secondary amines and amides	C–N stretch	Secondary amines and amides
620	C–N stretch	Secondary amines and amides	C–N stretch	Secondary amines and amides
1053	C–O–C stretch	Aromatic ethers and polysaccharides	C–O–C stretch	Aromatic ethers and polysaccharides
1109	–C–O	Terpenoids, flavones	Not present	
1250	C–H stretch and O–H deform. of carboxyl groups and bending of N–H bond	Amide III	C–H stretch and O–H deform. of carboxyl groups and bending of N–H bond	Amide III
1375	C–N stretch	Aromatic amines I, II	C–N stretch	Aromatic amines I, II
1516	C=C	Aromatic chain in carboxylic acids from patulein	C=C	Aromatic chain in carboxylic acids from patulein
1628	C=O bond	Luteolin	C=O bond	Luteolin
1737	C=O bond	Luteolin	C=O bond	Luteolin
2827	C–H stretch	Aliphatic group of glucosides	C–H stretch	Aliphatic group of glucosides
2926	C–H stretch	Aliphatic group of glucosides	C–H stretch	Aliphatic group of glucosides
3400	O–H stretch and N–H stretch	Phenols and amides	O–H stretch and N–H stretch	Phenols and amides

**Fig. 4:**
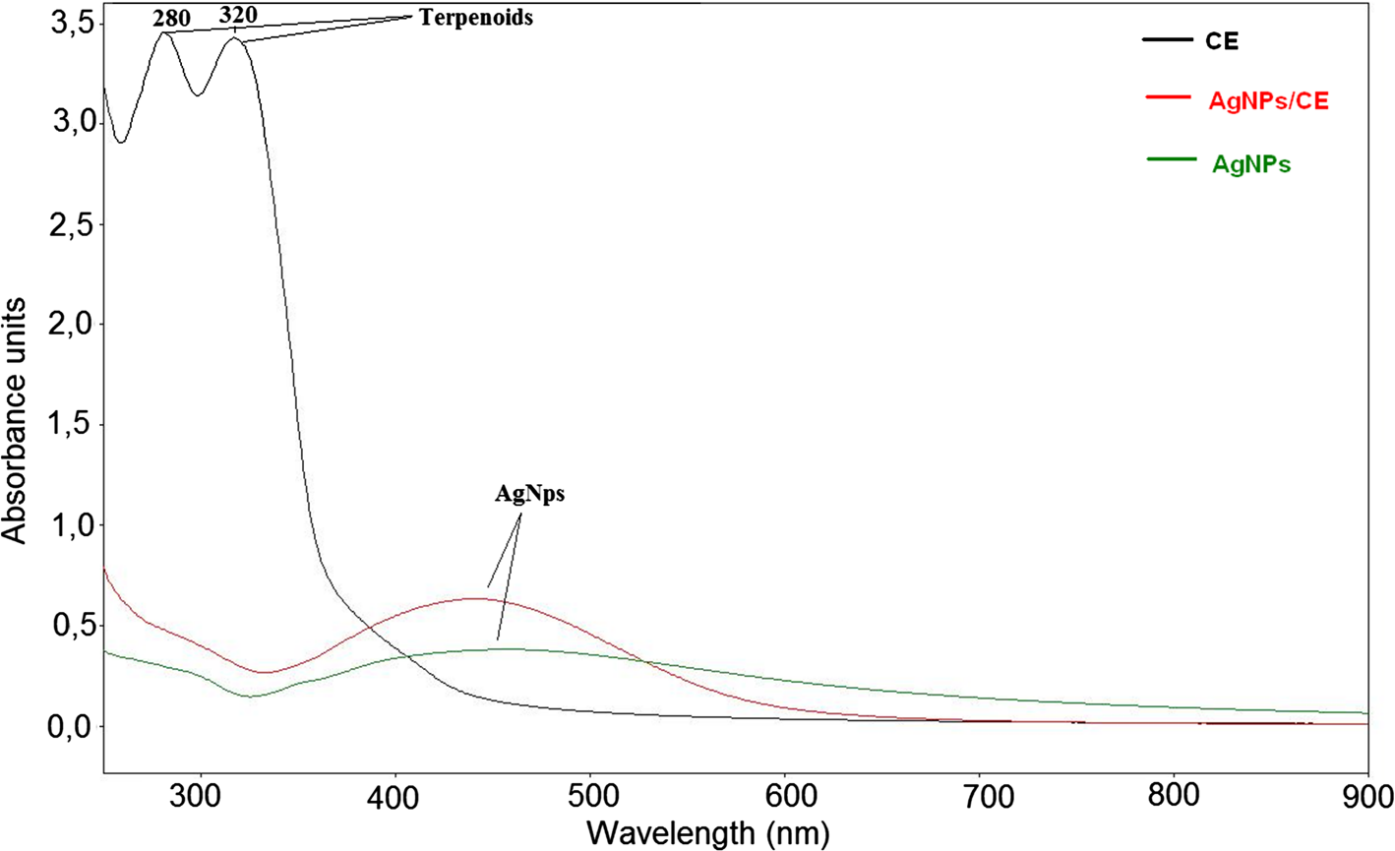
UV-vis spectra for: CE (*black*) showing the two peaks corresponding to terpenoids, for AgNPs/CE (*red*) and AgNPs/G (*green*)—only one peak from silver is visible (color figure online)

Figure 4 shows the UV–vis spectra of the CE (black), AgNPs/CE (red) and AgNPs (green) suspensions. On the spectrum of CE two peaks are present at 280 and 320 nm corresponding to terpenoid [37–40]. These peaks disappear in the spectra measured for AgNPs/CE and AgNPs/G. For these suspensions peaks at 425 nm originating from Ag nanoparticles are observed [41].

### Antibacterial properties

**Fig. 5:**
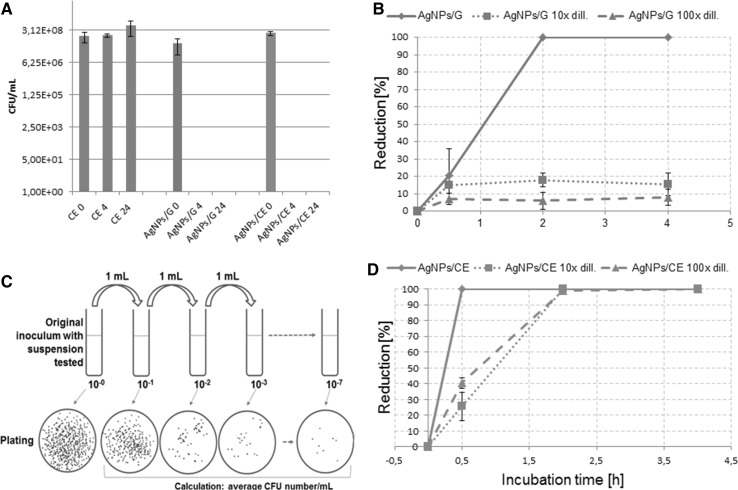
**a** Antibacterial test of camomile extract (CE), plant extract with silver nanoparticles (AgNPs/CE) and pure nanoparticles synthesized with glucose (AgNPs/G) against *S. aureus*. ** b** The percentage of microorganism (*S. aureus*) reduction exposed to the tested nanosuspensions: AgNPs/G (*above*) and AgNPs/CE (*below*). ** c** The flowchart for the evaluation of colony forming unit (CFU)

The antibacterial impact of the camomile synthesized Ag nanoparticles was assessed. A comparison between the antibacterial properties of pure camomile extract, pure silver particles and Ag particles synthesized in the camomile extract was performed according to the CLSI protocol. This experiment showed that the CE sample has little bacteriostatic effect (0.26 log reduction after 4 h exposure). Further, cultivation does not show any inhibition of the bacteria growth (Fig. 5a), on the contrary, after a longer incubation time, increasing of the number of bacteria was observed. Conversely, total inhibition of bacteria growth was observed after exposure of cells to both silver containing nanosuspensions, regardless the incubation time.

Figure 5b shows the inactivation efficiency for the two different nanosuspensions. Using different concentrations of the nanomaterial suspensions, different cell responses are observed. Pure silver nanoparticles show weaker degree of bacteria inhibition, especially, when comparing tenfold or hundredfold suspension dilution (upper part of Fig. 5b). The maximum percentage of reduction is 17.9 and 8.1 % for tenfold or hundredfold diluted AgNPs/G, respectively. Only concentrated AgNPs/G exhibit a strong inhibition effect on the growth of *S. aureus*, as a 100 % reduction is observed after 2 h of incubation. On the contrary, the nanosolution of AgNPs/CE, is much more antibacterial (bottom part of Fig. 5c). All tested concentrations have an impact on the bacteria growth: a total reduction of the bacteria after 2 h of exposure to all types of nanosuspensions, is observed. Summarizing, the antibacterial study shows that AgNPs/CE has excellent activity against *S. aureus*. The bacterial growth was limited for all AgNPs/CE samples, independently on their concentration.

**Fig. 6:**
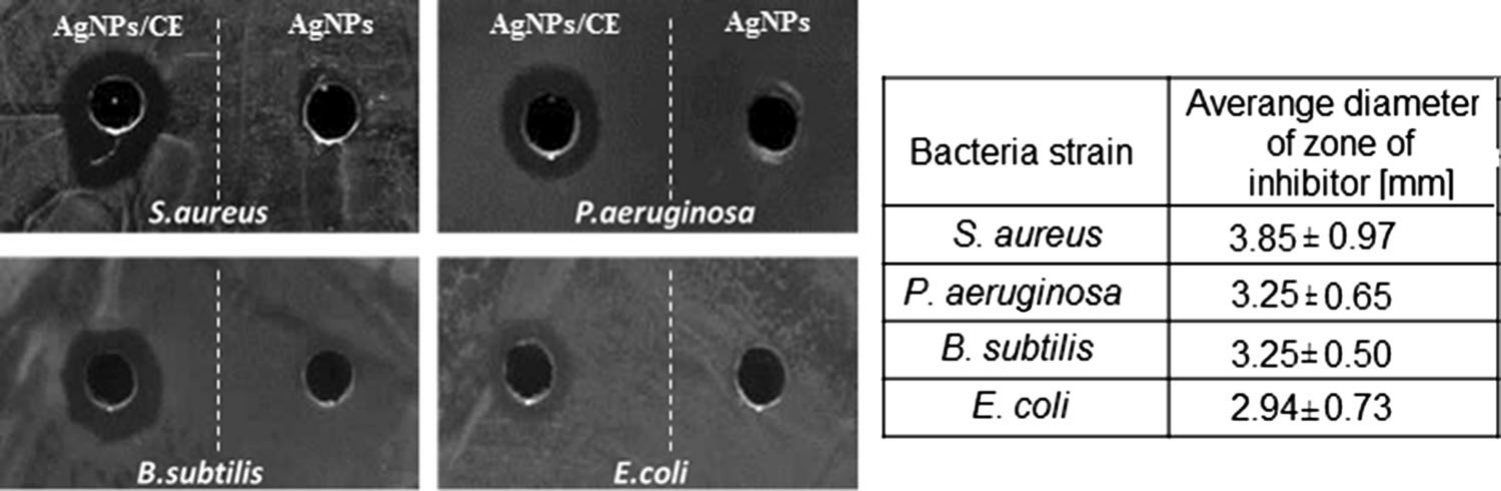
The mean zone of inhibition against various bacteria strains using agar diffusion method. The wells contain: camomile extract synthesized silver nanoparticles (AgNPs/CE)—*left side* or pure nanoparticles (AgNPs/G)—*right side*, respectively

We have extended our studies to the evaluation of the nanosolutions impact on different bacterial strains. It was estimated based on the zone of inhibition test. The obtained results of qualitative antibacterial diffusion tests, Fig. 6, showed that AgNPs/CE have an effect on all tested microorganisms, gram-positive and negative. The diameters of the average inhibition zones were 3.85, 3.25, 3.25 and 2.94 mm for *S. aureus*, *P. aeruginosa*, *B. subtilis* and *E. coli*, respectively. For the AgNPs/G nanosolution, no inhibition was observed; only a 1 mm zone was detected for *S. aureus*.

**Fig. 7:**
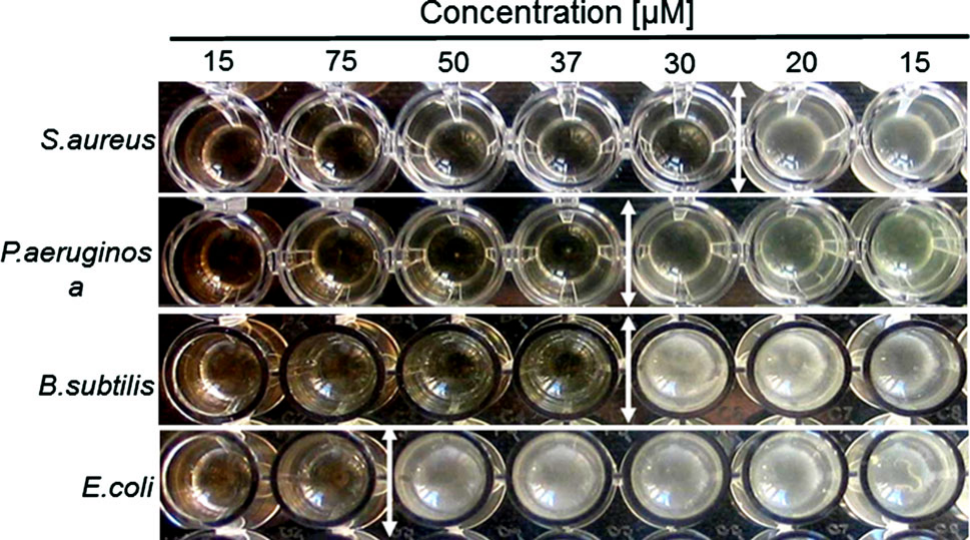
MICs of nanomaterials against bacterial strains. Different concentrations of nanosolutions, were estimated from the concentration of silver nitrate, which was used as precursor, and were exposed to *S. aureus*, *P. aeruginosa*, *B. subtilis* and *E. coli*. *White bars* on the photograph denote the MICs for this experiment

To confirm the antibacterial activity of green-synthesized Ag nanoparticles, we defined the minimum inhibitory concentration (MIC) for the two gram-positive and two gram-negative bacteria, which was defined as the lowest concentration of AgNPs/CE, at which the strain cannot produce visible growth after overnight culturing. The results, showed that silver nanoparticles synthesized with camomile extract exhibit a high antibacterial activity, Fig. 7. These materials have the MICs for four strains ranging from 20 to 50 µM. *Staphylococcus aureus* was found as the most sensitive strain, and was able to growth only when exposed to 20 µM of AgNPs/CE, while *E. coli* seems to be the most resistant after exposure to AgNPs/CE with MIC of 50 µM.

## Discussion

STEM structural analyses of the camomile extract showed that it is composed of highly porous globules formed by aggregation and agglomeration processes, Fig. 2a, b. Hence, they have a very developed surface area, guaranteeing an easy attachment of the Ag NPs and making them a perfect support for the Ag nanoparticles. In the EDX maps, Fig. 2c, d, oxygen and phosphorus are detected. Both elements are expected to be present in camomile, as its chemical constituents include among others terpenoids, flavones flavonoids, apigenin and alpha-bisabolol. Indeed, all of these compounds are composed of hydrogen, oxygen and carbon. The latter element is also present as the support foil on the Cu grid, and is thus, detected by EDX as background in all the samples. Hydrogen is an element, which cannot be detected by EDX. Terpenoids in their chemical formula contain phosphorus additionally to C, H and O, Fig. 2m. Therefore, it was decided to map phosphorous and oxygen.

The structural analysis of AgNPs/CE showed Ag nanoparticles located on globular structures, similar to the ones observed in pure camomile. This morphology proves that indeed the Ag nanoparticles were reduced in the camomile extract. The Ag particles are uniformly distributed on the camomile globules, making their very large surface available for antibacterial activity. Terpenoids are believed to be the surface-active molecules that stabilize nanoparticles [41] preventing the Ag NPs from agglomeration. Surprisingly, phosphorus is detected in the EDX maps on the Ag particles rather than, as expected, on the camomile globules, Fig. 2h. This might be explained combing this observation with the FTIR results showing the involvement of terpenoids in the reduction of Ag^+^ ions. The involvement of phenolics, proteins, terpenoids and other reducing agents in the synthesis of metal nanoparticles has been speculated [37]. The reduction from Ag^+^ ions to silver nanoparticles (Ag^0^) with terpenoids may involve the conversion of C–O group of the terpenes to –C–O group [40]. It is also possible that the terpenoids play a role in the reduction of metal ions by oxidation of aldehydic groups in the molecules to carboxylic acids [37]. The experimentally observed presence of phosphorus on the surface of Ag in the EDX maps strongly suggests that indeed terpenoids could be adsorbed on the surface of metal nanoparticles, possibly by interaction through carbonyl groups or *π*-electrons in the absence of other strong ligating agents in sufficient concentration. Moreover, the peak visible in the FTIR spectrum of CE at 1109 cm^−1^ and the double peak in the UV–vis spectrum of CE at 280 and 320 nm corresponding to –C–O group in terpenoids disappear in the FTIR and UV–vis spectra of the AgNPs/CE, respectively [35]. The measurements performed by these three experimental techniques confirmed that terpenoids are indeed adsorbed on the AgNPs surface preventing their agglomeration. The difference in intensity and width of the peaks for AgNPs/CE and AgNPs/G spectra is directly related to the size of the Ag particles, which are more than six times larger in pure AgNPs suspension compared to the AgNPs/CE.

The above-discussed results strongly suggest that terpenoids act as capping agents during synthesis of Ag nanoparticles. Capping agents are ionic species, small molecules, or macromolecules that can selectively bind to different types of facets on a nanocrystal to change their specific surface free energies and in this way their area proportions [46]. Introducing a capping agent into a reaction solution, the type of facet stabilized by the capping agent will exhibit a lower specific surface free energy. This will result in the formation of nanocrystals with a shape favoring a certain type of facet, as the capping agent chemisorbed on a facet will prevent the deposition of atoms onto this facet [46, Fig. 5]. The AgNPs/CE on the STEM image, Fig. 2f, have an octahedron-like shape. Thus, camomile terpenoids seem to act as a capping agent for {111} facets promoting the nanoparticle growth along the <100> directions. The proportion of the Ag {100} facets is reduced during growth leading consequently to the formation of octahedrons enclosed only by {111} facets.

The test of survival of *S. aureus* bacteria cells exposed to the tested suspensions lead to the conclusion, that both Ag containing nanosuspensions are extremely toxic to bacteria. Other authors studied Ag nanoparticles synthesized with camomile exposed to *E. coli* strain, showing high toxicity of Ag on bacteria [42, 43] which is consistent with our results. To estimate the antibacterial activity of our nanosuspensions the difference in toxicity between CE, AgNPs/Ce and AgNPs/G was verified. Among the tested suspensions, only pure extract did not exhibit any bactericidal effect. This is consistent with the data obtained by Kaviya, who synthesized silver NPs using citrus sinensis peel extract, instead of the camomile extract [43]. Ghosh et all postulated that if silver nanoparticles and some natural metabolite have bactericidal potential, these components could be utilized together. Their synergistic action should enhance the antibacterial effect [44]. This effect was compared for pure AgNPs/G and AgNPs/CE suspensions, depending on their concentration.

The results of the cytotoxicity study show that the antibacterial activity of AgNPs/CE is much more efficient against pathogenic bacteria than the one of AgNPs/G. In comparison to AgNPs/CE, the smaller antibacterial effect (observed in all antibacterial assays) of pure AgNPs/G is due to their large size combined with strong agglomeration. This suggests that the high microbial activity of AgNPs/CE originates from the synergistic effect of substances such as phenolics and flavonoids present in the camomile extract and the Ag particles. Indeed, Gogoi and co-authors obtained similar bactericidal results using green synthesis of silver nanoparticles based on alcoholic flower extract of *Nyctanthes arbortristis* [44, 45]. This hypothesis is confirmed also by the MIC test as well as zone of inhibition Fig. 7. In both cases, AgNPs/CE suspension clearly affected the bacterial growth.

Electron microscopy analysis of the AgNPs/CE showed that the Ag nanoparticles have sizes between 2 and 12 nm and are well dispersed on the globular camomile support, prohibiting their agglomeration. This dispersion combined with the small size allows these Ag particles to exploit their entire active surface—the smaller the particle the higher its activity. The synthesis of Ag in camomile extract containing terpenoids allowed preventing agglomeration of the Ag particles, which is a common problem for nanosized particles reducing their activity. The AgNPs/G synthesized without camomile is agglomerated, Fig. 2 column III, what reduces considerably their active surface, which would explain their lower antibacterial activity compared to the AgNPs/CE. These findings clearly demonstrate that it is indeed possible to have a much greener way to synthesize AgNPs without compromising their antibacterial properties. Thus, plant extracts may prove to be a good alternative to obtain such NPs with improved antibacterial and antiviral properties for antibacterial applications.

## Conclusions

Structural and antibacterial properties of camomile extract synthesized Ag nanoparticles were assessed and compared with the performance of pure camomile extract and pure Ag nanoparticles synthesized with glucose. Indeed, it was experimentally proven that AgNPs synthesized with CE are uniformly distributed on porous globular structures constituting the camomile support. The dispersion of these AgNPs combined with their size between 2 and 12 nm allows them to use their whole active surface, which is thus much larger than the one of pure AgNPs/G with sizes exceeding 50 nm. In the AgNPs/CE suspension, the EDX maps of phosphorus, a component of terpenoids, showing its distribution on the Ag particles, combined with the FTIR and UV–vis spectra, where the peak originating from terpenoids disappears, leads to the conclusion that terpenoids play an important role in the reduction of Ag^+^ ions to Ag metallic nanoparticles. Moreover, terpenoids are most probably adsorbed on the metal surface as surface-active molecules, preventing the AgNPs from agglomeration. The antibacterial activity of the three suspensions was compared. The CE sample did not show any bactericidal effect. Conversely, both solutions containing AgNPs showed an antibacterial performance. In the % of reduction, zone of inhibition and minimum inhibitory concentration tests, the AgNPs/CE solution performed outstandingly the best, killing all bacteria strains in short time. This study shows that camomile, with its porous globular structure constitutes a perfect support for AgNPs offering simultaneously a synergistic antibacterial effect. Moreover, the used green way of synthesis of AgNPs/CE allows reducing the environmental impact, as camomile extract is non-toxic to living things and the environment.
